# Drugs for COVID-19: An Update

**DOI:** 10.3390/molecules27238562

**Published:** 2022-12-05

**Authors:** Jessica Ceramella, Domenico Iacopetta, Maria Stefania Sinicropi, Inmaculada Andreu, Annaluisa Mariconda, Carmela Saturnino, Federica Giuzio, Pasquale Longo, Stefano Aquaro, Alessia Catalano

**Affiliations:** 1 Department of Pharmacy, Health and Nutritional Sciences, University of Calabria, Arcavacata di Rende, 87036 Rende, Italy; 2 Departamento de Química, Universitat Politècnica de València, Camino de Vera s/n, 46022 Valencia, Spain; 3 Unidad Mixta de Investigación UPV-IIS La Fe, Hospital Universitari i Politècnic La Fe, Avenida de Fernando Abril Martorell 106, 46026 Valencia, Spain; 4 Department of Science, University of Basilicata, 85100 Potenza, Italy; 5 Department of Chemistry and Biology, University of Salerno, Via Giovanni Paolo II, 132, 84084 Fisciano, Italy; 6 Department of Pharmacy-Drug Sciences, University of Bari “Aldo Moro”, 70126 Bari, Italy

**Keywords:** antibiotics, anticoagulants, antivirals, COVID-19, interleukin inhibitors, JAKs inhibitors, immunomodulators, NSAIDs, SARS-CoV-2

## Abstract

The severe acute respiratory syndrome coronavirus 2 (SARS-CoV-2) was the seventh known human coronavirus, and it was identified in Wuhan, Hubei province, China, in 2020. It caused the highly contagious disease called coronavirus disease 2019 (COVID-19), declared a global pandemic by the World Health Organization (WHO) on 11 March 2020. A great number of studies in the search of new therapies and vaccines have been carried out in these three long years, producing a series of successes; however, the need for more effective vaccines, therapies and other solutions is still being pursued. This review represents a tracking shot of the current pharmacological therapies used for the treatment of COVID-19.

## 1. Introduction

The ongoing COVID-19 pandemic, caused by SARS-CoV-2, poses a serious threat to public health worldwide. In addition to the massive number of deaths (globally, on the 14 November 2022, there have been 630,832,131 confirmed cases of COVID-19, including 6,584,104 deaths, reported by WHO [1]), many other factors and behaviors have changed following the pandemic. Several variants have been described for this virus [2] mainly due to SARS-CoV-2 adaptation in the human host, evidenced by the emergence of new viral lineages, termed variants of concern (VOCs), which include Alpha, Delta and Omicron. Most of the severe cases of COVID-19 have been due to the Delta variant that has been dominant since May 2021 [3]. Next, the recently identified Omicron variant has rapidly displaced the Delta one, becoming the most common SARS-CoV-2 variant. The failure of the global COVID-19 vaccination and the emerging new SARS-CoV-2 Omicron lineages BA.4 and BA.5 are crucial factors in the ongoing pandemic [4]. The very latest BA.2.75.2 variant exhibits extensive escape from neutralizing antibodies [5]. Moreover, the post-COVID syndrome, that is the permanency of symptoms in patients who have recovered from COVID-19, is now becoming a second pandemic, evidencing stiffness, headache, muscle and joint pain and generally perceived deterioration in patients’ health [6,7]. Pharmaceutical companies and researchers worldwide are proposing different strategies to mitigate the diffusion and transmission of the virus and develop a cure for the produced disease. The therapies used for COVID-19 can be classified into two categories: therapies acting directly against the virus or the host [8]. Effective therapies against SARS-CoV-2 exploit the use of antivirals, antimalarials, antibiotics, corticosteroids and immunomodulatory drugs. These compounds interfere with SARS-CoV-2 replication, regulate the inflammatory response generated by the immune system, and finally treat the symptoms. In the early stages of the disease, when the active viral replication is predominant, antiviral drugs are used, while for the following stages, in which the inflammatory process prevails, the use of immunomodulatory agents is more appropriate [9]. With the drugs used to treat COVID-19, several clinical studies are ongoing to find new effective treatments for this disease, overcoming multidrug and antimicrobial resistance [10]. However, for this overwhelming pandemic, “there aren’t always right answers, but some answers are wrong”, as reported by the American Board of Internal Medicine [11] regarding the promotion of misinformation by physicians. For instance, the experience taught us that some drugs first chosen for COVID-19 treatment have been demonstrated to produce scarce or even self-defeating effects, receiving WHO negative recommendations [12]. In this review, we aimed to report the most-used drugs, summarized in Table 1, to treat COVID-19.

## 2. Characteristics of Coronaviruses

**Figure 1:**
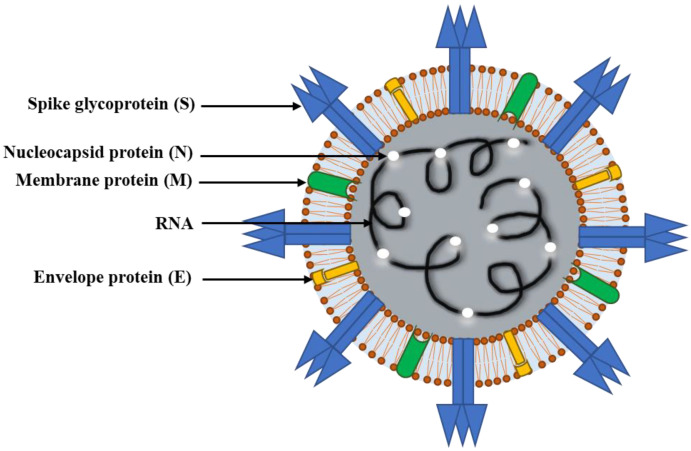
Structure of SARS-CoV-2.

Coronaviruses (CoVs) are viruses belonging to the Coronoviridae family, which can cause diseases with different symptoms, ranging from the common cold to severe respiratory syndromes. They are enveloped, positive-stranded RNA viruses, with a genome size from 26 to 32 kilobases (Kb). They are classified into four genera: Alpha CoV (α-CoV), Beta CoV (β-CoV), Gamma CoV (γ-CoV) and Delta CoV (Δ-CoV). The α-CoV and β-CoV infect mammals, such as bats, pets and humans, while γ-CoV and Δ-CoV mainly infect birds. The major structural proteins of SARS-CoV-2 include the spike (S), membrane (M), envelope (E) and nucleocapsid (N) proteins (Figure 1). The S glycoprotein comprises two functional subunits responsible either for binding to the host cell receptor: S1 subunit including the receptor-binding domain (RBD) and S2 subunit for the viral fusion to the cellular membranes. The entry of SARS-CoV-2 into the host cell depends on the binding of the S1 unit to a cell receptor, which facilitates the attachment of the virus to the surface of the target cells [13,14]. As demonstrated, SARS-CoV-2 uses the angiotensin-converting enzyme 2 (ACE2) as a receptor to gain the entry into the human cells [15], then the virus enters by endocytosis thanks to the proteolytic cleavage of the S protein by a specific protease, the trans-membrane serine protease type 2 (TMPRSS2). In the host cell cytoplasm, the viral RNA is then released for genome replication and viral protein synthesis, followed by the assembly and release of virions that infect the neighboring cells, spreading the infection [16]. Thus, SARS-CoV-2 engages the angiotensin-converting enzyme 2 (ACE2) as an entry receptor and the TMPRSS2 for the activation of protein S [17]. Moreover, Main protease (M^pro^), also known as the 3-chymitrypsin-like protease (3CL^pro^), and papain-like protease (PL^pro^) have been suggested to be essential for viral replication and several drugs are directed to inhibit them [18]. Other antiviral drugs target the RNA-dependent RNA polymerase (RdRp) as well, which plays a crucial role in virus replication [8].

## 3. Antiviral Drugs

The available antiviral drugs may act on different viral mechanisms, from the prevention of the virus entry into the host cells to the suppression of the various replication and assembly steps within the cells [19]. Particularly, the main targets of antiviral drugs are represented by the spike protein [20], ACE2 [21], TMPRSS2 [22], M^pro^ (or 3CL^pro^) [23] and PL^pro^ [24]. A series of antivirals adopted for the treatment of COVID-19 patients will be discussed below.

### 3.1. Remdesivir (Veklury)

Remdesivir is a prodrug belonging to the class of nucleotide analogs, first developed for the treatment of Ebola virus disease and then for various single-stranded RNA viruses, including CoVs s. Remdesivir is an adenosine analog that inhibits RdRp activity, which plays a crucial role in virus replication [25]. After entering the cell, the drug is metabolized to its three major derivatives: nucleotide monophosphate, nucleotide triphosphate and nucleoside. The nucleoside triphosphate acts as an analog of adenosine triphosphate (ATP) and competes with the natural substrate for the incorporation into the newly synthetized RNA, catalyzed by the RdRp, provoking early chain termination [26]. On 1 May 2020, the United States Food and Drug Administration (FDA) authorized the use of remdesivir as emergency treatment for hospitalized COVID-19 patients, aged 12 or older. The use of this drug is not recommended in the case of mild or moderate patients; however, for those who need respiratory support, its use reduces the time of recovery and the risk of disease progression [26]. Remdesivir intravenous dosages are 200 mg in the first 24 h, followed by 100 mg per day for the following four days [27]. However, remdesivir did not produce significant effects on COVID-19 patients already ventilated and it showed little effect against death and/or progression to ventilation [28]. 

### 3.2. Favipiravir

Favipiravir is an antiviral drug firstly used in Japan in 2014 for the treatment of influenza; then, it showed activity against a wide range of RNA viruses, including the Ebola virus. It is a prodrug analog of guanine and its active form inhibits the viral RdRp, preventing genome replication. In March 2020, favipiravir was approved by the National Medical Products Administration of China as the first anti-COVID-19 drug, after a clinical trial that demonstrated its efficacy with minimal side effects [29]. The drug causes a decrease in viral load within 4 days, with a clinical improvement of up to 88% in patients with mild to moderate COVID-19 [30]. In addition, favipiravir is also used in Hungary, India, Korea, Poland, Portugal, Russia, Serbia, Thailand and Turkey [31]. In 2021, Shinkai et al. [32] conducted a clinical trial employing COVID-19 patients with moderate pneumonia, without oxygen therapy and within 10 days of fever onset; they were treated with 1800 mg of favipiravir twice a day on day 1 and then 800 mg twice a day for up to 13 days. The results of this study indicated that favipiravir diminished the time of patients’ clinical improvement and suggested that, for those at high risk of aggravation, its early administration could be beneficial, even though the onset of adverse effects, including hyperuricemia, must be considered. In Italy and other countries, however, some studies have shown that favipiravir had no significant beneficial effect on mortality among mild to moderate COVID-19 patients [31]. Finally, AlQahtani et al. [33] reported a similar therapeutic utility of favipiravir compared to hydroxychloroquine and the standard therapy in mild to moderate COVID-19 patients, even though the favipiravir therapy was safe and seemed to increase the viral clearance. However, the use of favipiravir may be controversial. Hassanipour et al. [34] reported that favipiravir possibly exerted no significant beneficial effect in the term of mortality in patients with mild to moderate COVID-19.

### 3.3. Lopinavir/Ritonavir

Lopinavir (LVP) is a protease inhibitor of the human immunodeficiency virus 1 (HIV-1), metabolized via cytochrome P450 isoenzyme 3A4, and is the major enzyme involved in the metabolism of protease inhibitors. It is generally used in association with ritonavir, an effective inhibitor of the cytochrome P450 isoenzyme 3A4, in order to increase its plasma half-life. LVP is able to inhibit viral replication by inactivating the proteases M^pro^ and PL^pro^ [26]. The literature data illustrate different results on lopinavir/ritonavir combination (LPV/r) treatment for COVID-19 [33], and their association was found to be effective in some in vitro studies and trials in patients with SARS-CoV-2 [35]. Ye et al. (2020) [36] reported that LPV/r reduced the body temperature and restored normal physiological function, compared with the control group, without inducing toxicity. On the other hand, a following randomized study, which involved 199 patients with severe COVID-19, showed that LPV/r therapy did not produce positive results compared to the standard protocol [37]. Patients were divided into two groups: the first group of patients were given 400 mg of lopinavir and 100 mg of ritonavir twice daily, for up to 14 days, along with the standard care; the second group received only the standard care. The addition of LPV/r to the standard therapy did not improve the clinical outcomes nor significantly reduce mortality [26]. The study by Wen et al. (2020) [38] demonstrated that LPV/r did not promote virus negative conversion and clinical improvement in COVID-19 patients [39]. An updated systematic review examined whether the use of LPV/r could be more favorable compared to the standard therapies regarding the length of stay, time for positive-to-negative conversion of SARS-CoV-2 and mortality in hospitalized patients with COVID-19, concluding that LPV/r had no significant advantage and also registered a significant increase in the occurrence of side effects [40]. However, Elmekaty et al. [41] showed that early treatment with LPV/r is associated with a faster clinical improvement and/or virological clearance than the darunavir/cobicistat therapy in patients with COVID-19 pneumonia, and that the safety profile was quite comparable. Finally, Wong et al. [42] discourage the use of LPV/r for pediatric COVID-19 cases because of their negative clinical outcomes, reporting a longer time for clinical improvement, seroconversion, hospital discharge, together with a higher risk of a hyperinflammation.

### 3.4. Molnupiravir (Lagevrio)

Molnupiravir is a prodrug that has proven efficacy in the treatment of infections caused by RNA viruses, such as influenza viruses and CoVs [43]. After being distributed in the tissues, it is transformed into the pharmacologically active ribonucleoside triphosphate *N*-hydroxycitidine triphosphate, which is incorporated into the RNA by RdRp, causing a viral error catastrophe due to the accumulation of mutations within the viral genome and resulting, ultimately, in the inhibition of virus replication [25]. On 4 November 2021, the British Drug Agency MHRA (Medicines and Healthcare Products Regulatory Agency) approved molnupiravir as the first oral antiviral for the treatment of COVID-19, mostly for patients at risk of serious illness [44]. Efficacy studies established that oral administration of molnupiravir reduces the risk of severe disease by about 50% compared to the placebo in patients with mild to moderate disease [45]. The recommended dosage of molnupiravir was 800 mg every 12 h for 5 days. Its action is guaranteed if administered immediately after the diagnosis of COVID-19 within 5 days of the onset of symptoms [46]. Jeong et al. [47] reported that combined treatment with nirmatrelvir (20 mg/kg) and molnupiravir (20 mg/kg) in SARS-CoV-2 lethally infected K18-hACE2 transgenic mice produced an effective inhibition of SARS-CoV-2 replication and increased the survival rates up to 80% in comparison with the mice treated with only nirmatrelvir or molnupiravir. Moreover, the synergic treatment reduced the clinical severity score, tissue damage, and viral distribution with respect to the animals administered with the monotherapy. Summing up, the combined use of nirmatrelvir and molnupiravir ameliorate both morbidity and mortality. Next, Wong et al. [48] conducted a retrospective cohort study aimed at the evaluation of molnupiravir and nirmatrelvir plus ritonavir efficacy on non-hospitalized patients with COVID-19, during the Hong Kong’s SARS-CoV-2 wave in which the omicron BA.2.2 variant was predominant (January–June 2022). The use of 800 mg twice daily for 5 days allowed a reduction in the risk of death by 24% with molnupiravir, compared with the use of any oral antivirals, and a reduced risk of hospitalization. Additionally, the use of ventilation was reduced in molnupiravir-treated patients compared with the controls and the early administration of either molnupiravir or nirmatrelvir plus ritonavir reduced the risks of disease progression and death. 

### 3.5. Paxlovid (Nirmatrelvir/Ritonavir)

Paxlovid is a therapeutic combination of two drugs: nirmatrelvir, which inhibits the 3-chymotrypsin-like protease (3CLpro) of SARS-CoV-2, and ritonavir, an effective inhibitor of the cytochrome P450 isoenzyme 3A4, which increases the plasma half-life of nirmatrelvir. In one clinical study, 1219 participants were divided into two groups, one of which received paxlovid, given orally every 12 h for five consecutive days, and the other half received placebo [49]. In patients treated with paxlovid, within five days of symptom onset, a reduction in COVID-19-related hospitalization or death was observed [50]. On 22 December 2021, the Food and Drug Administration (FDA) granted emergency use authorization (EUA) for paxlovid in adults and children with mild and moderate COVID-19 at increased risk of progression. Ganatra et al. [51] conducted a comparative retrospective cohort study, in a period from December 2021 and April 2022, on non-hospitalized and vaccinated patients that, even so, developed COVID-19. The study indicates that treatment with paxlovid, within five days after diagnosis, produced a reduction in multisystem symptoms (e.g., respiratory tract infections, cardiac arrhythmia, gastrointestinal issues) and complications. Additionally, Zheng et al. [52] indicated that the paxlovid treatment is effective and safe for patients with COVID-19, diminishing both the hospitalization and mortality rate by 78%, and that there was not a significant difference of rebound and adverse effects between the paxlovid-treated and the control groups. Zhong et al. [53] suggested that the administration of paxlovid (300 mg nirmatrelvir and 100 mg ritonavir for 5 days) to elderly Chinese patients infected by the SARS-CoV2 omicron variant reduces the viral nucleic acid shedding time. The study period ranged from April to May 2022, and no heavy secondary effects or death were recorded, with only a bitter mouth feeling in 26.42% of cases. However, the drug is not recommended for patients with severely impaired liver or kidney function, and recognized side effects include nausea, diarrhea and increased blood pressure [44]. The suggested dose for patients with normal renal function is 300 mg of nirmatrelvir combined with 100 mg of ritonavir taken orally twice daily for five days [50]. 

### 3.6. Simeprevir

Simeprevir is an NS3/4A protease inhibitor for use in HCV genotypes 1 and 4. It is orally administered and achieves high virological cure rates. Recently, it has been reported that simeprevir is an especially promising drug for treating COVID-19 because it potently reduces SARS-CoV-2 viral load by multiple orders of magnitude [54]. 

## 4. Antimalarial Drugs

### Chloroquine and Hydroxychloroquine

Chloroquine and hydroxychloroquine are drugs classically used in the treatment of autoimmune diseases, such as rheumatoid arthritis and systemic lupus erythematosus, and then repurposed as COVID-19 treatment [55]. Their antiviral activity is mainly due to their ability to increase the endosomal pH which, in turn, prevents the fusion of the viral membrane with the endosome, leading to interference with the uncoating of the virus [56]. For this reason, both chloroquine and its hydroxyl derivative were widely used in many countries during the first wave of COVID-19. A first clinical trial in China reported significant improvements in over 100 cases of COVID-19 patients treated with chloroquine [57]. In other studies, it has also been shown that the combination of hydroxychloroquine with the antibiotic azithromycin increases the effectiveness of the treatment, compared to the administration of hydroxychloroquine alone [33]. However, significant adverse effects have limited the administration of these drugs in COVID-19 patients, including nausea, vomiting, diarrhea, and ventricular arrhythmias [58]. Furthermore, some clinical studies showed that hydroxychloroquine and chloroquine were not able to reduce the mortality rate in hospitalized patients compared to standard care [59]. Therefore, based on scientific data, on June 15, 2020, the FDA determined that hydroxychloroquine and chloroquine were not recommended for the treatment of COVID-19 [26,60].

## 5. Antibiotics

### Azithromycin

Azithromycin is an antibiotic belonging to the macrolide family, acting against Gram-positive and Gram-negative bacteria through the inhibition of protein synthesis. In addition to its antibacterial activity, it has shown interesting antiviral and immunomodulatory properties [61]; for instance, it was found to be active in vitro against Ebola and Zika viruses and prevented severe viral infections of the respiratory tract [62]. The antiviral effect of the drug is due to its ability to increase the endosomal pH, thus blocking the endocytosis and therefore the uncoating of the virus [63]. In vitro studies have also shown good antiviral activity against SARS-CoV-2 and the ability to decrease pro-inflammatory cytokine and chemokine secretion [61]. Many clinical studies on the effects of azithromycin in the treatment of SARS-CoV-2 have been conducted in combination with the above-discussed hydroxychloroquine. A first clinical study, conducted in France, engaged 36 patients that were divided into three groups: one group, consisting of 14 people, was treated with hydroxychloroquine alone; the second group (6 people) was administered with a combination of hydroxychloroquine and azithromycin; the last, the control group (16 people), did not receive any drug treatment. The obtained results showed that the combination of azithromycin/hydroxychloroquine was able to reduce the viral load more efficiently than the single treatment [61]. However, in other clinical studies, the combined administration of azithromycin and hydroxychloroquine did not lead to any improvement in the clinical status of the patients [64,65]. Furthermore, the administration of hydroxychloroquine/azithromycin in hospitalized patients has been linked to an increased risk of adverse cardiac events, such as the prolongation of the QT interval and ventricular arrhythmia [66]. Based on all the clinical studies performed, the Italian Medicines Agency (AIFA) has stated that the use of azithromycin, alone or in combination with hydroxychloroquine, for the treatment of COVID-19 patients is not recommended, unless bacterial infections do not occur [62].

## 6. Interleukine Inhibitors

### 6.1. Anakinra

Anakinra, an interleukin-1 (IL-1) receptor antagonist, is an anti-inflammatory drug mainly used for the treatment of rheumatoid arthritis [67]. The use of this drug in patients affected by COVID-19 produces positive effects in controlling the cytokine storm, consequently reducing the risk of acute respiratory distress syndrome (ARDS) and respiratory and organ failure [68]. Various studies have been carried out on the use of this drug in COVID-19 patients and, interestingly, in one of them, Anakinra reduced the need for mechanical ventilation and mortality in patients with severe COVID-19 [69]. A further study, conducted in patients with COVID-19 and ARDS, showed that Anakinra (5 mg/kg twice daily) could be safely used in order to improve respiratory function [67]. In a recent study, the effectiveness of Anakinra in improving respiratory condition and in decreasing the use of mechanical ventilation and the hospitalization duration was confirmed. Moreover, this study evidenced the absence of toxicity related to the use of Anakinra in patients with critical conditions [70]. A meta-analysis highlighted the ability of Anakinra to act as a strong anti-inflammatory agent in the treatment of COVID-19 patients, reducing hyper-inflammatory markers, including C-reactive protein concentrations, serum ferritin, and D-dimer levels [71].

### 6.2. Tocilizumab (RoActemra)

Tocilizumab is a humanized monoclonal antibody that selectively binds the interleukin-6 (IL-6) receptor, preventing the cytokine from binding its receptor. It is normally used for the treatment of rheumatoid arthritis and cytokine release syndrome [72] and has also been employed in the treatment of COVID-19 after its authorization by AIFA on 3 April 2020 in a phase III study evaluating its safety and efficacy. Then, numerous studies suggested the efficacy of tocilizumab in the treatment of patients suffering from COVID-19. For example, in a study involving 100 patients with COVID-19, tocilizumab induced a rapid and sustained clinical improvement [73]. In other studies, this drug reduced the length of hospitalization, the need for mechanical ventilation, and the intensive care unit admission. Additionally, add-on therapy with tocilizumab reduced mortality and decreased the risk of progression from severe to critical illness compared to standard care [74]. A recent meta-analysis demonstrated that tocilizumab reduced the risk of progression to mechanical ventilation and death in COVID-19 patients with elevated inflammation and low levels of oxygen saturation. Moreover, the effect of this drug was amplified by the concurrent administration of corticosteroids [75] which seemed to have a potential role in reducing mortality even in the elderly population [76].

## 7. Janus Kinase (JAKs) Inhibitors

### Baricitinib

Baricitinib is a reversible inhibitor of Janus kinases 1 and 2 (JAK1 and JAK2), intracellular enzymes involved in the transmission of the cytokine signals and growth factors involved in hematopoiesis and immune response [49]. It was approved in more than 65 countries for the treatment of patients with moderate to severe rheumatoid arthritis [77], then it was reported to influence, as well, the cellular entry of SARS-CoV-2, due to its ability to inhibit the AP2-associated protein kinase, a known regulator of endocytosis [78]. Several clinical studies, conducted to assess the role of baricitinib in the management of patients suffering from COVID-19, showed the ability of this drug to reduce the levels of inflammatory markers and improve oxygenation. The daily administration of baricitinib for 6 days was found to be effective in the treatment of severe COVID-19, showing improvements in clinical and analytical parameters without significant side effects [79]. A randomized open-label trial indicated that baricitinib decreased the risk of death by about one-fifth in hospitalized COVID-19 patients; however, the amount of benefit was less than that indicated by previous studies [80]. A further study found that the combination of baricitinib with remdesivir reduced the recovery times and accelerated improvement in clinical status in hospitalized patients with COVID-19, compared to those who received remdesivir alone [81]. 

## 8. Corticosteroids

### Dexamethasone

Corticosteroids are among the most widely used anti-inflammatory drugs for the treatment of inflammatory and immune diseases. Several studies confirmed the beneficial effect of corticosteroids in patients with COVID-19 [82]; however, an inappropriate dose of corticosteroids in COVID-19 patients can cause various moderate and long-term adverse effects, including increased insulin resistance and cardiovascular risk, bacterial infections, glucose metabolism disturbance, thrombotic complications and allergic reactions. Dexamethasone, a synthetic corticosteroid, is a glucocorticoid used to treat asthma, allergic reactions, arthritis and other autoimmune diseases [83]. A retrospective observational study showed that a high dose of dexamethasone induced a significant improvement in clinical and laboratory parameters in hospitalized COVID-19 patients under the hyperinflammatory phase [84]. In another study, the effectiveness of low doses of dexamethasone was compared to the usually used high dosage [82]. In particular, 6 mg of dexamethasone were administered to COVID-19 patients once daily for ten days, resulting in a reduction in the mortality rate. Despite this, and considering the various toxic effects, on 2 September 2020, the World Health Organization (WHO) issued a guideline on the use of dexamethasone and other corticosteroids for the treatment of patients with COVID-19, recommending their use only in severe and critical patients [83]. 

## 9. Anticoagulants

### Low Molecular Weight Heparin (LMWH)

Low molecular weight heparin (LMWH) is used for the prophylaxis of venous thromboembolism [85]. Although it is not a drug with a specific antiviral action, it has been included among those that can be used in the treatment of COVID-19 and, in particular, the management of the progressive alteration of some inflammatory and coagulative parameters in the advanced phase of the disease [73]. In fact, increased levels of fibrin degradation fragments, such as D-dimer, have been observed in COVID-19 patients, which is indicative of the coagulation pathway activation, with the risk of pulmonary and venous thromboembolism. LMWH can be used in the initial phase of COVID-19as a prophylaxis for venous thromboembolism, if pneumonia, which leads to hypomobility in bedridden patients, is present. It is also used in the advanced stage of the disease in hospitalized patients with hyperinflammation, to prevent the thrombotic phenomena [73]. In addition to its anticoagulant effect, LMWH has a potential antiviral activity, being able to interfere with the viral spike protein and preventing the virus binding to the cell surface. Moreover, it possesses systemic anti-inflammatory properties due to its ability to decrease the levels of pro-inflammatory mediators, causing a decrease in the migration and activation of immune cells [86]. 

## 10. Non-Steroid Anti-Inflammatory Drugs (NSAIDs)

Non-steroidal anti-inflammatory drugs (NSAIDs) are used to relieve pain, reduce inflammation and for their antipyretic effect, being able to inhibit the enzymes cyclooxygenase-1 (COX-1) and cyclooxygenase-2 (COX-2), which mediate the production of prostaglandins. COX-1 is constitutively expressed in most cells, while COX-2 expression is induced by inflammatory stimuli, and both metabolize the arachidonic acid into the prostaglandin H2, which can be converted into various prostaglandins [87]. Prostaglandins are lipid molecules that play various biological roles in homeostasis and inflammatory responses, such as the regulation of immune responses and the integrity of the gastrointestinal barrier. Since the development of severe inflammation, pain and fever are part of COVID-19 patients’ symptoms, NSAIDs are often used [88]. Unfortunately, the inhibition of prostaglandins’ production by NSAIDs can influence the pathogenesis of COVID-19, and many concerns have been raised about the use of NSAIDs to treat the COVID-19 symptoms [89]. In particular, in March 2020, a study highlighted that the activity of the angiotensin 2 converting enzyme (ACE-2), the cellular entry receptor exploited by SARS-CoV-2, can be increased by ibuprofen [90]. Furthermore, the use of NSAIDs could compromise the immune response and delay the resolution of the disease itself. However, numerous studies have shown that ibuprofen and meloxicam, two NSAIDs commonly used for the treatment of COVID-19 symptoms, had no effect on the ACE2 enzyme expression, viral entry or replication itself [89]. Therefore, the current recommendations of some guidelines indicate that these drugs should not be contraindicated in patients with COVID-19 [88].

## 11. Recent Studies for New Drugs

In view of the ongoing mutation of SARS-CoV-2 (such as Omicron), new clinical studies have been addressed to small-molecule anti-CoV drugs, considering the convenience and flexibility of oral administration and the large production capacity. These are summarized in Table 2. Some clinical advances in the development of small-molecule drugs targeting SARS-CoV-2 3CL^pro^ have been reported by Hu et al. [91]. The noncovalent, reversible oral nonpeptidic SARS-CoV-2 3CL protease inhibitor clinical candidate, namely S-217622 developed by Shionogi, has been deeply studied [92]. This compound was discovered via virtual and biological screening of an in-house compound library, and optimization of the hit compound using a structure-based drug design strategy [93]. It is under evaluation in a phase III clinical trial (NCT05305547) and is a prospective oral therapeutic option for COVID-19. Moreover, new viral and cellular pathways are currently being investigated as potential targets to develop effective therapy to stop the pandemic [94]. Recently, the nanoligomer treatment SBCoV207, which has been already validated for its ability in mitigating uncontrolled immune response in SARS-CoV-2 infection [95], has shown a high bioavailability and biodistribution to the lungs and produced no toxicity in mice at 10 mg/kg, when administered via intranasal, intraperitoneal, or intravenous routes [96]. Opaganib (aka ABC294640), a drug targeting the sphingolipid metabolism and used for the treatment of inflammatory diseases, also exhibited an antiviral activity towards different viruses, including SARS-CoV-2. Particularly, opaganib reduced the mortality of patients with moderately severe COVID-19 by about 62%, due to its ability to suppress SARS-CoV-2 infection and replication by inhibiting three enzymes in sphingolipid metabolism: sphingosine kinase-2, dihydroceramide desaturase and glucosylceramide synthase. Thus, this drug may represent a safe alternative to remdesivir and dexamethasone with both antiviral and anti-inflammatory properties [97]. Interestingly, G-quadruplexes specific ligands, namely 5,10,15,20-tetrakis-(*N*-methyl-4-pyridyl)porphine (TMPyP4), showed better antiviral effects than remdesivir on the Vero E6 cells, Syrian hamster and human angiotensin-converting enzyme 2 (hACE2) transgenic mouse model of SARS-CoV-2 infection, with no significant toxicity [98]. Most importantly, this study provided an alternative strategy for COVID-19 treatment by targeting the secondary genomic structures of SARS-CoV-2, paving the way to the rational design and synthesis of new safer agents.

## 12. Conclusions

COVID-19 has upset the lives of everyone worldwide. At the end of 2019 in Wuhan, China, the health authorities confirmed dozens of pneumonia cases and a new virus belonging to the coronavirus family, identified as SARS-CoV-2. In the recent past, CoVs were already responsible for two epidemics, SARS and MERS, in China (2003) and the Middle East (2021), respectively. SARS-CoV-2 rapidly spread throughout China and the rest of the world and the WHO officially declared a pandemic in March 2020. Like other respiratory diseases, SARS-CoV-2 infection can cause mild symptoms such as colds, sore throats, coughs, and fever, but in some cases, COVID-19 occurs in a more severe form, with pneumonia and respiratory distress syndrome. SARS-CoV-2 has changed a lot since its first appearance, giving rise to several variants. Since the beginning of the COVID-19 pandemic, intense research has been carried out to identify, in the shortest possible time, some pharmacological agents capable of blocking the entry of SARS-CoV-2 or inhibiting its replication. Several drugs are used, including antiviral and antimalarial agents, antibiotics, immunomodulators, angiotensin II receptor blockers, bradykinin B2 receptor antagonists and corticosteroids. A quick and effective approach was drug repositioning, namely the uncovering of new indications of some approved compounds, used to treat some diseases or that failed or that have been abandoned for whatever reason, to use in a different disease. Among the many potential drug candidates, remdesivir, lopinavir/ritonavir, and chloroquine (or hydroxychloroquine), have received an increased scientific attention, but only remdesivir has been approved by the FDA for the treatment of patients with COVID-19, although its clinical efficacy is still controversial. The main objective for managing COVID-19 is to provide new antiviral drugs with a direct action against SARS-CoV-2 and develop vaccines with a broader and more lasting protection. Furthermore, in order to face the current and future global challenges and prevent other viral epidemics from turning into pandemics, there is a real and urgent need to develop specific antiviral and antibacterial drugs. Unfortunately, the battle against the COVID-19 is not over yet; however, vaccinations, exposure to the virus and the availability of new and effective pharmacological treatments, useful for preventing severe forms of the disease and reducing the number of infections, may represent a glimmer of hope towards the retention of the pandemic.

## Figures and Tables

**Table 1:** Drugs used to treat COVID-19.

Structure	Name	Activity
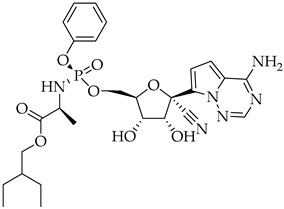	Remdesivir	Antiviral viral RdRp inhibitor
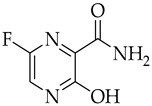	Favipiravir	Antiviral viral RdRp inhibitor
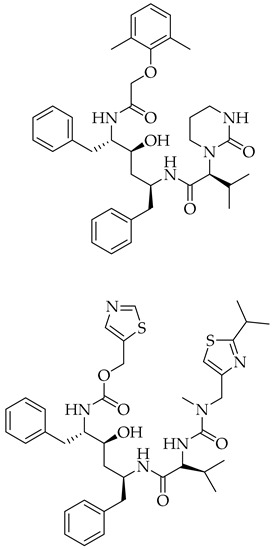	Lopinavir + Ritonavir	Antiviral viral protease inhibitor
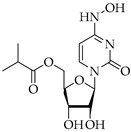	Molnupiravir	Antiviral viral RdRp inhibitor
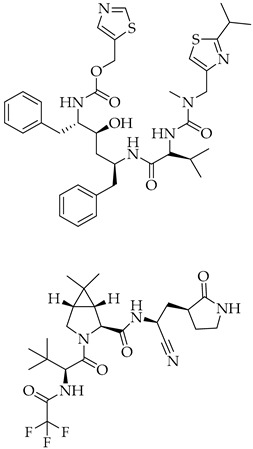	Paxlovid (ritonavir + nirmatrelvir)	Antiviral viral protease inhibitor
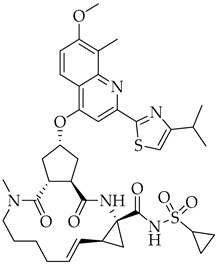	Simeprevir	Antiviral NS3/4A protease inhibitor
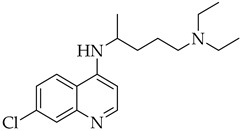	Chloroquine	Antimalarial increase in endosomal pH → block of the uncoating of the virus
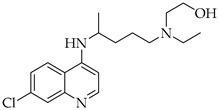	Hydroxychloroquine	Antimalarial increase in endosomal pH → block of the uncoating of the virus
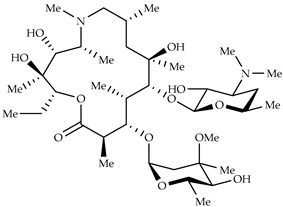	Azithromycin	Antimicrobial increase in endosomal pH → block of the uncoating of the virus
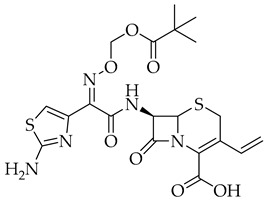	Anakinra	Interleukin-1 inhibitor
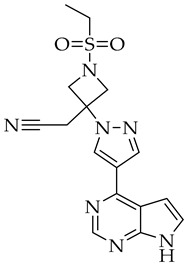	Baricitinib	JAK inhibitor
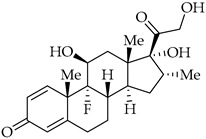	Dexamethasone	Corticosteroid
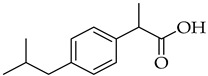	Ibuprofen	NSAID
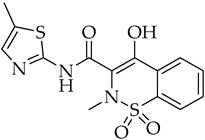	Meloxicam	NSAID

**Table 2:** New drugs for the treatment of COVID-19.

Structure	Name	Activity
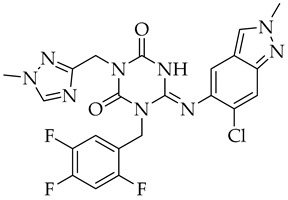	S-217622	SARS-CoV-2 3CL protease inhibitor
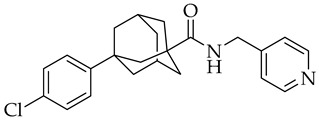	Opaganib	Sphingosine kinase-2, dihydroceramide desaturase and glucosylceramide synthase inhibitor
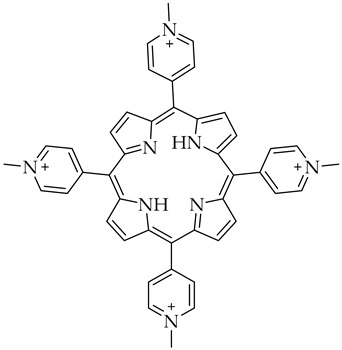	5,10,15,20-tetrakis-(*N*-methyl-4-pyridyl)porphine (TMPyP4)	G-quadruplexes specific ligand

## Data Availability

Not applicable.
